# Targeting circulating high mobility group box-1 and histones by extracorporeal blood purification as an immunomodulation strategy against critical illnesses

**DOI:** 10.1186/s13054-023-04382-0

**Published:** 2023-02-28

**Authors:** Yupei Li, Yu Chen, Tinghang Yang, Kaixi Chang, Ningyue Deng, Weifeng Zhao, Baihai Su

**Affiliations:** 1 Department of Nephrology, West China Hospital, Sichuan University, Chengdu, China; 2 State Key Laboratory of Polymer Materials Engineering, College of Polymer Science and Engineering, Sichuan University, Chengdu, China; 3 Med-X Center for Materials, Sichuan University, Chengdu, China; 4 Med+ Biomaterial Institute of West China Hospital, Sichuan University, Chengdu, 610041 China

**Keywords:** High mobility group box-1, Histone, Extracorporeal blood purification, Inflammation, Sepsis, Critical illness

## Abstract

Both high mobility group box-1 (HMGB1) and histones are major damage-associated molecular patterns (DAPMs) that mediate lethal systemic inflammation, activation of the complement and coagulation system, endothelial injury and multiple organ dysfunction syndrome in critical illnesses. Although accumulating evidence collectively shows that targeting HMGB1 or histones by their specific antibodies or inhibitors could significantly mitigate aberrant immune responses in multiple critically ill animal models, routine clinical use of such agents is still not recommended by any guideline. In contrast, extracorporeal blood purification, which has been widely used to replace dysfunctional organs and remove exogenous or endogenous toxins in intensive care units, may also exert an immunomodulatory effect by eliminating inflammatory mediators such as cytokines, endotoxin, HMGB1 and histones in patients with critical illnesses. In this review, we summarize the multiple immunopathological roles of HMGB1 and histones in mediating inflammation, immune thrombosis and organ dysfunction and discuss the rationale for the removal of these DAMPs using various hemofilters. The latest preclinical and clinical evidence for the use of extracorporeal blood purification to improve the clinical outcome of critically ill patients by targeting circulating HMGB1 and histones is also gathered.

## Introduction

Critical illnesses are commonly associated with immune dysregulation, characterized by concurrent hyperinflammation and immune suppression [1]. It has been well established that endotoxins are major inflammatory mediators that trigger the release of both proinflammatory and anti-inflammatory cytokines and organ injury in critically ill patients, especially those with sepsis, during the past two decades [2–4]. Accordingly, endotoxins and cytokines are widely recognized as the main therapeutic targets during extracorporeal blood purification (EBP) sessions for the immunomodulation of critical illnesses [4, 5]. Currently, EBP uses a series of hemofilters to remove hydrophilic or hydrophobic solutes through the mechanism of convection, diffusion or adsorption, and the solute removal spectrum of a hemofilter is significantly dependent on its own membrane/adsorbent structure and treatment dose [6]. Multiple hemofilters, such as Toraymyxin hemofilter (Toray Industries, Tokyo, Japan), the CytoSorb hemofilter (CytoSorbents Corporation, New Jersey, USA) and the oXiris hemofilter (Baxter, Meyzieu, France), have been used to treat critically ill patients in current clinical practice aiming to eliminate endotoxins and/or cytokines despite the lack of solid evidence of survival benefit [3, 7]. The failure of EBP therapies to improve survival in these patients could be attributed to the inadequate timing for treatment initiation, inadequate patient selection, inadequate therapeutic target selection and insufficient clearance of inflammatory mediators [3, 5]. Instead of the removal of cytokines that are downstream mediators in the immune cascade, the elimination of upstream proinflammatory mediators, such as the pathogen, activated host immune cells and especially damage-associated molecular patterns (DAMPs), could be more suitable therapeutic targets for EBP therapies in the early phase of critical illnesses.

Recent reviews have demonstrated the multiple roles of DAMPs, which are produced or released by damaged and dying cells, in sterile inflammation and tissue repair after they are sensed by various pattern recognition receptors [8, 9]. Currently, an increasing number of endogenous host-derived molecules, including S100 proteins, heat shock proteins, high mobility group box 1 protein (HMGB1), circulating histones and glycans such as heparan sulfate, are considered DAMPs [10]. Among them, HMGB1 and histones are abundantly studied because they significantly mediate lethal systemic inflammation, complement and coagulation activation, endothelial injury and organ dysfunction in various critical illnesses, such as sepsis [11, 12], acute liver failure [13], pancreatitis [14, 15], multiple trauma [16] and severe COVID-19 [17, 18]. Accumulating evidence collectively suggests that high levels of serum HMGB1 and histones significantly correlate with disease severity and mortality in critically ill patients [16, 19–23]. Accordingly, targeting HMGB1 and histones to alleviate inflammation and tissue injury might be an important immunomodulation strategy against these critical diseases. To be gratified, the last decade has witnessed the rapid development of diverse histone-neutralizing agents and HMGB1 antagonists to alleviate the immunopathological processes induced by such DAMPs and thus to improve the outcome of sepsis [12, 24], liver failure [25, 26] and pancreatitis [27, 28] animal models. For instance, heparin, the most commonly used histone-neutralizing agent, not only prevents histone-mediated coagulation activation and organ injury in mice [29, 30] but also significantly alleviates the HMGB1-induced inflammatory response [31] and lung endothelial dysfunction through the P38-GSK3β-snail signaling pathway [32]. However, routine use of heparin in critically ill patients is not always available because the inherent anticoagulant activity of heparin unfortunately increases the risk of fatal bleeding in those with coagulopathy. None of the currently available anti-histone or anti-HMGB1 agents have been approved for immunomodulation in the management of critical illnesses by any clinical guideline due to the lack of evidence. Most recently, EBP using multiple hemofilters, such as Cytosorb [33], AN69ST membrane [34] and heparin-functionalized Seraph-100 blood filter [35], was found to adsorb circulating HMGB1 and histones in vitro and in vivo. It is noteworthy that the use of such EBP devices in patients with critical illness has great potential to mitigate HMGB1/histone-mediated inflammation, immune thrombosis and organ injury without the administration of traditional anti-histone or anti-HMGB1 agents.

In this review, we aim to clarify the immunopathological roles of HMGB1 and histones in critical illness, discuss the rationale for the removal of DAMPs using different hemofilters and summarize the latest preclinical and clinical evidence in this field. We also provide perspectives for the future design and clinical evaluation of novel hemofilters for the removal of DAMPs in the blood of critically ill patients. Of note, our review exclusively discusses the effect of EBP with various hemofilters on the removal of DAMPs, and we unfortunately fail to further demonstrate the immunomodulatory effect of such hemofilters on immune cell functions, a major component of the immune response, due to a lack of evidence.

## Overview of the immunopathological roles of HMGB1 and histones in critical illness

### HMGB1

HMGB1, a multifunctional and highly conserved nucleoprotein with two positively charged DNA-binding regions and a negatively charged tail, has been implicated in the pathogenesis of multiple critical illnesses, including sepsis [11], acute liver failure [13, 36] and severe trauma [37]. HMGB1 is usually released by activated macrophages as an alarmin during prolonged inflammation [38, 39]. HMGB1 signals through Toll-like receptors (TLRs), the receptor for advanced glycation end products, the NF-κB-inflammasome and/or the CXCL-12-CXCR4-NF-κB-inflammasome axis and induces inflammation and organ damage even in the absence of infection [11, 40, 41]. Circulating HMGB1 also interacts with the complement cascade to augment sterile inflammation in critical illness [42, 43]. In both experimental sepsis models and in sepsis patients, the levels of HMGB1 in the plasma are markedly increased, which positively correlates with disease severity [11]. Emerging evidence shows that acute liver failure patients/animals have high concentrations of circulating HMGB1, which can contribute to multiple organ injuries and mediate gut bacterial translocation [13]. A recent observational study by Yang et al. further found significantly increased plasma concentrations of HMGB1 in combat casualties on arrival to the hospital correlated positively with blood inflammatory mediators [44]. An in vivo study showed that CX-01 (2-O,3-O-desulfated heparin) with < 5% anticoagulant activity significantly inhibited systemic HMGB1 activity, decreased local and systemic inflammatory responses, and reduced tissue and organ damage in a blast injury-induced trauma rat model [44]. Many studies have claimed a positive correlation between extracellular HMGB1 and severe acute pancreatitis (SAP) severity in recent decades [15]. Moreover, HMGB1 also contributes to the development of MODS secondary to SAP, and blockade of HMGB1 by its neutralizing antibody could significantly attenuate the development of SAP and SAP-associated organ dysfunction [28, 45]. These results collectively suggest that circulating HMGB1 is a promising immunomodulation target in critical illness.

### Circulating histones

Beyond HMGB1, histones are another group of basic nucleoproteins mainly derived from neutrophil extracellular traps that contribute to a dysregulated inflammatory response, activation of the coagulation system and organ dysfunction in sepsis [12], liver failure [13, 26], trauma [16, 46], SAP [14, 47], acute respiratory distress syndrome (ARDS) [48, 49] and COVID-19 [18]. Circulating histones, also known as extracellular histones, significantly activate TLR-dependent and NLPR3 inflammasome pathways to trigger the host innate immune response, resulting in the activation of the NF-κB pathway and subsequent release of proinflammatory cytokines such as TNF-α, IL-6, IL-1β and IL-18 [12, 50, 51]. Histones also exert potent cytotoxicity to vascular endothelial cells in a dose-dependent manner by binding to phospholipid–phosphodiester bonds in cell membranes and thus altering membrane permeability and initiating calcium ion influx [16, 52]. Meanwhile, histones mediate the disruption of endothelial barrier function and vascular permeability by inducing oxidative stress and pyroptosis in endothelial cells [53, 54], disrupting cell–cell adherens junctions [55, 56], upregulating the expression of adhesion molecules ICAM1, VCAM1, and E-selectin [55, 57, 58], and impairing the endothelial glycocalyx [59, 60]. Furthermore, histone-induced proinflammatory cytokine release and vascular endothelial injury will unfortunately disrupt the fine balance and cross talk between coagulant, anticoagulant and inflammatory pathways to trigger, amplify and propagate disseminated intravascular coagulation (DIC) [61]. Recent studies have also shown that histones in the blood circulation significantly increase tissue factor expression in endothelial cells and monocytes [62, 63], increase lytic cell death of macrophages and phosphatidylserine exposure [64], reduce endogenous anticoagulant activity of vascular endothelium [65], and induce platelet activation, aggregation and consumption [66, 67] to mediate DIC. It is noteworthy that such deleterious effects of histones can be blocked by heparin, and thus, developing anti-histone agents (heparin or heparinoids) holds great potential for reducing MODS and improving the survival of critically ill patients. Our previous publications have also summarized the immunopathological roles of histones in mediating sterile inflammation, endothelial dysfunction, coagulation activation and organ dysfunction and concluded that circulating histones are promising immunomodulation targets in multiple critical illnesses [12, 68].

## Removal of HMGB1 and histones by hemofiltration or hemoperfusion

### Hemofiltration

#### AN69ST membrane

The AN69ST membrane is a surface-treated polyacrylonitrile-co-methallyl sulfonate membrane that has excellent adsorption capacities for cytokines owing to its anionic hydrogel structure [69–71]. For instance, Shiga et al. found that the AN69ST membrane significantly decreased the mean blood IL-6 and lactate levels in patients with septic shock after a 72-h continuous hemodiafiltration therapy [71]. In a mono-compartmental in vitro model containing 100 μg of HMGB1 and 35 g of bovine albumin, it was shown that hemofiltration using the AN69ST membrane had superior adsorption capacity for HMGB1 than the polymethylmethacrylate, polyethersulfone and polysulfone membranes [72]. The reduction ratios of HMGB1 by the AN69ST membrane during 60 min and 360 min were 97.3% and 99.3%, respectively, with a high HMGB1 clearance of 60.8 ± 5.0 mL/min at 15 min [72]. More recently, another in vitro study by Tomoyuki et al. showed that the AN69ST membrane primarily removes 93.6–99.3% of HMGB1 in bovine serum albumin-spiked substitution fluid by bulk adsorption without any sign of adsorption saturation. Meanwhile, the HMGB1 clearances by the AN69ST membrane at 0, 10 and 30 min were consistently high over the 360-min course (52.8–60.3 mL/min) with a high HMGB1 adsorption capacity of 700 μg [34]. Therefore, hemofiltration with the AN69ST membrane could eliminate lethal levels of both HMGB1 and proinflammatory cytokines in septic patients. Further clinical studies are urgently needed to evaluate the impact of the use of the AN69ST membrane to remove HMGB1 on patient-centered clinical outcomes. As the polyethyleneimine layer of the AN69ST membrane can also adsorb heparin to allow heparin priming before use [7], it is also of clinical significance to further determine whether the use of the AN69ST hemofilter is able to remove positively charged histones by the primed heparin layer from the blood of critically ill patients receiving continuous renal replacement therapy.

#### Polymethylmethacrylate membrane

The polymethylmethacrylate (PMMA) membrane was first developed by Toray Industries, Inc., and has been widely used in both chronic dialysis and sepsis patients [73]. Since the 2010s, it has been well established that continuous hemodiafiltration with the PMMA membrane could remove various proinflammatory cytokines from the bloodstream mainly by adsorption rather than convection and diffusion [74, 75]. A small-size clinical study even observed a higher 28-day survival in 43 septic shock patients with acute kidney failure who received continuous hemofiltration with the PMMA membrane than in those with a polyacrylonitrile membrane (83.3% vs. 30.8%) [73]. Furthermore, the PMMA membrane also showed a considerable HMGB1 adsorption clearance of 25.8 ± 4.8 mL/min at 15 min, nearly half of the adsorption capacity of the AN69ST membrane [72]. More recently, Ryusuke et al. investigated the effect of pore structures and sizes of three PMMA membrane fibers on their HMGB1 adsorption capacities and found that the obtained HKT fiber with a surface pore size of 41.4 nm exhibited the highest HMGB1 adsorption capacity of 1879 ± 414 ng/g among the three fibers tested [76]. The biocompatibility and HMGB1 adsorption performance of the HKT column were further studied in a d-galactosamine-induced acute liver failure swine model. The ratio of HMGB1 at the outlet versus the inlet was less than one-third throughout the 4-h hemoperfusion with the HKT column, suggesting high-performance plasma HMGB1 reduction using the porous HKT fiber for extracorporeal hemoperfusion in large animals. However, the potential survival benefit of HKT column hemoperfusion should be determined in other types of liver failure animal models because the animals in the present d-galactosamine models are too severe to survive long enough.

### Hemoperfusion

Hemoperfusion is a modality for EBP in which solute removal is achieved by binding molecules to adsorbent materials [3]. Unlike indirect solute removal by hemodialysis membranes through the mechanism of convection and diffusion, hemoperfusion allows direct adsorption of specific substances using hemoperfusion cartridges with large surface areas and high adsorptive capacities [3]. Furthermore, insufficient substance adsorption by the abovementioned AN69ST or PMMA membranes might unfortunately lead to membrane fouling and negatively affect the removal of small- to medium-sized solutes during EBP sessions. Accordingly, hemoperfusion cartridges with specific or nonspecific solute removal spectra are of great clinical significance in the management of critically ill patients without the need for traditional renal replacement.

#### Polymyxin B hemoperfusion cartridge

The polymyxin B hemoperfusion cartridge (PMX, Toray Industries, Tokyo, Japan) is an adsorbent cartridge composed of polystyrene fibers bound to polymyxin B that is marketed in Japan and Europe for the removal of endotoxins during sepsis and septic shock. It is well established that endotoxin levels decrease in vitro within minutes after starting PMX hemoperfusion [77]. However, the survival benefit of PMX hemoperfusion in septic patients remains controversial. Data from the EUPHAS trial demonstrated that PMX treatment was associated with a mortality benefit [28-day mortality: 32% in the PMX group vs. 53% in the control group; adjusted hazard ratio 0.36, 95% confidence interval (CI) 0.16–0.80] and a hemodynamic benefit in 64 patients with abdominal septic shock [78]. In contrast, the ABDOMIX trial enrolling 243 septic shock patients with peritonitis reported an insignificant difference in 28-day mortality of 27% in the PMX group and 19.5% in the control group (odds ratio 1.5872, 95% CI 0.8583–2.935, *p* = 0.14) [79]. More recently, the EUPHRATES trial included 450 adult critically ill patients with septic shock and an endotoxin activity assay level of 0.60 or higher to determine the effect of PMX hemoperfusion on 28-day mortality of septic patients. The results showed that PMX treatment was not associated with a significant difference in 28-day mortality among all participants (37.7% in the PMX group and 34.5% in the control group, relative risk 1.09, 95% CI 0.85–1.39, *p* = 0.49) [80]. For the first time, the EUPHRATES trial applied the endotoxin activity assay as a criterion to enroll patients, making PMX treatment potentially open to monitoring. A subsequent post hoc analysis of the EUPHRATES trial successfully demonstrated that PMX hemoperfusion significantly decreased 28-day mortality from 41.9 to 20% along with improved hemodynamics when the target population was narrowed to patients with endotoxin activity assay measurements between 0.6 and 0.9 [81]. Accordingly, the identification of appropriate recipients for PMX treatment by the endotoxin activity assay may have the potential to improve patient survival.

Beyond the elimination of endotoxin, PMX hemoperfusion also plays a vital role in the removal of HMGB1. As early as 2007, Sakamoto et al. demonstrated that HMGB-1 levels improved significantly in septic shock patients after successful PMX hemoperfusion in a retrospective study [82]. Likewise, another study enrolling 20 septic patients with ARDS found that direct hemoperfusion with a PMX column significantly decreased blood HMGB1 levels from 26.5 ± 12.5 to 2.8 ± 0.6 ng/mL [39]. Similar results were obtained in a later small-size study by the same research group in which the significant decrease in HMGB1 level was correlated with that in endotoxin [83]. Given that PMX is a medical device that aims to remove circulating endotoxin by adsorption, the reduction in circulating HMGB1 by PMX may be attributed to the blockade of the HMGB1-receptor for advanced glycation end-products axis through reduction of endotoxin and subsequent interleukin-6 [83].

#### Cytosorb adsorber

**Fig. 1:**
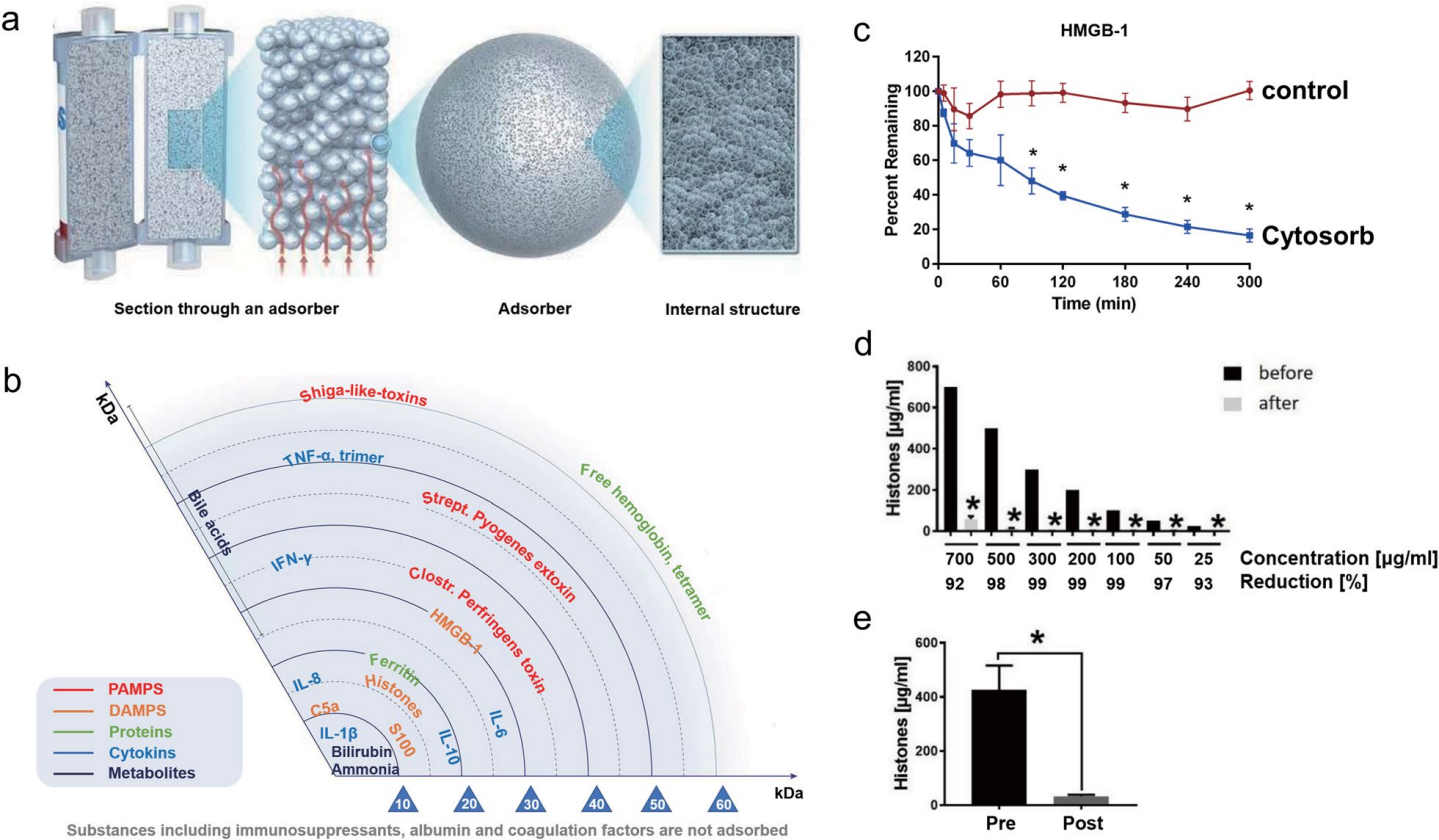
Structure, adsorption spectrum and HMGB1/histone adsorption performance of the Cytosorb® adsorber. **a** Microstructure of the Cytosorb® adsorber (©2022 Cytosorbents Europe GMBH); **b** Cytosorb adsorption spectrum (©2022 Cytosorbents Europe GMBH); **c** Adsorption of HMGB1 from whole blood with Cytosorb® adsorber or a control device ( © 2018 Gruda et al.); **d** Hemoadsorption of different concentrations of histones presented as percentage of reduction (%) after 6-h incubation with Cytosorb® adsorber (© 2020 Weber et al.); **e** Plasma histone levels of 22 multiple injured humans (µg/mL) in the Emergency room (pre) and after 6-h hemoadsorption (post) (© 2020 Weber et al.)

The Cytosorb adsorber has long been approved for the removal of cytokines, bilirubin, and myoglobin by hemoperfusion [84, 85]. The adsorber consists of a cylindrical cartridge filled with tiny, highly porous, hemocompatible polyvinylpyrrolidone-coated polystyrene-divinyl-benzene copolymer beads with a total surface area of > 40,000 m^2^, which significantly adsorbs hydrophobic cytokine molecules within the 5–55 kDa molecular weight range (see Fig. 1a and b) [86, 87]. In the last decade, the effect of EBP with Cytosorb adsorber on multiple critical illnesses, including infective endocarditis [88, 89], SAP [90], severe COVID-19 [91, 92], postcardiac arrest syndrome [93] and septic shock [94], has been extensively investigated in numerous RCTs and matched cohort studies. Although most of the related studies showed that the use of the Cytosorb hemofilter in severe patients with systemic cytokine storms considerably decreased serum levels of both pro- and anti-inflammatory cytokines, significant survival benefits of Cytosorb hemoadsorption have not been confirmed [85]. Recently, an in vitro experiment showed that Cytosorb® could not only reduce the levels of the cytokines MIP1-α, IL-6 and IFN-γ by 98 ± 4.0%, 91 ± 3.0% and 82 ± 15% in whole blood but also remove 83%-98% of DAMPs C5a, HMGB-1, procalcitonin, and S100-A8, which relies on a combination of pore capture and surface adsorption (see Fig. 1c) [87]. Subsequently, Weber et al. found that a 6-h hemadsorption procedure by the Cytosorb adsorber significantly decreased circulating histone levels in the blood of 22 humans with multiple injuries (see Fig. 1d and e) [33]. These studies collectively suggest that Cytosorb hemoperfusion may offer a therapeutic approach in critically ill patients via control of the massive release of various DAMPs, such as extracellular HMGB1 and histones.

#### Heparin-functionalized adsorbents

**Fig. 2:**
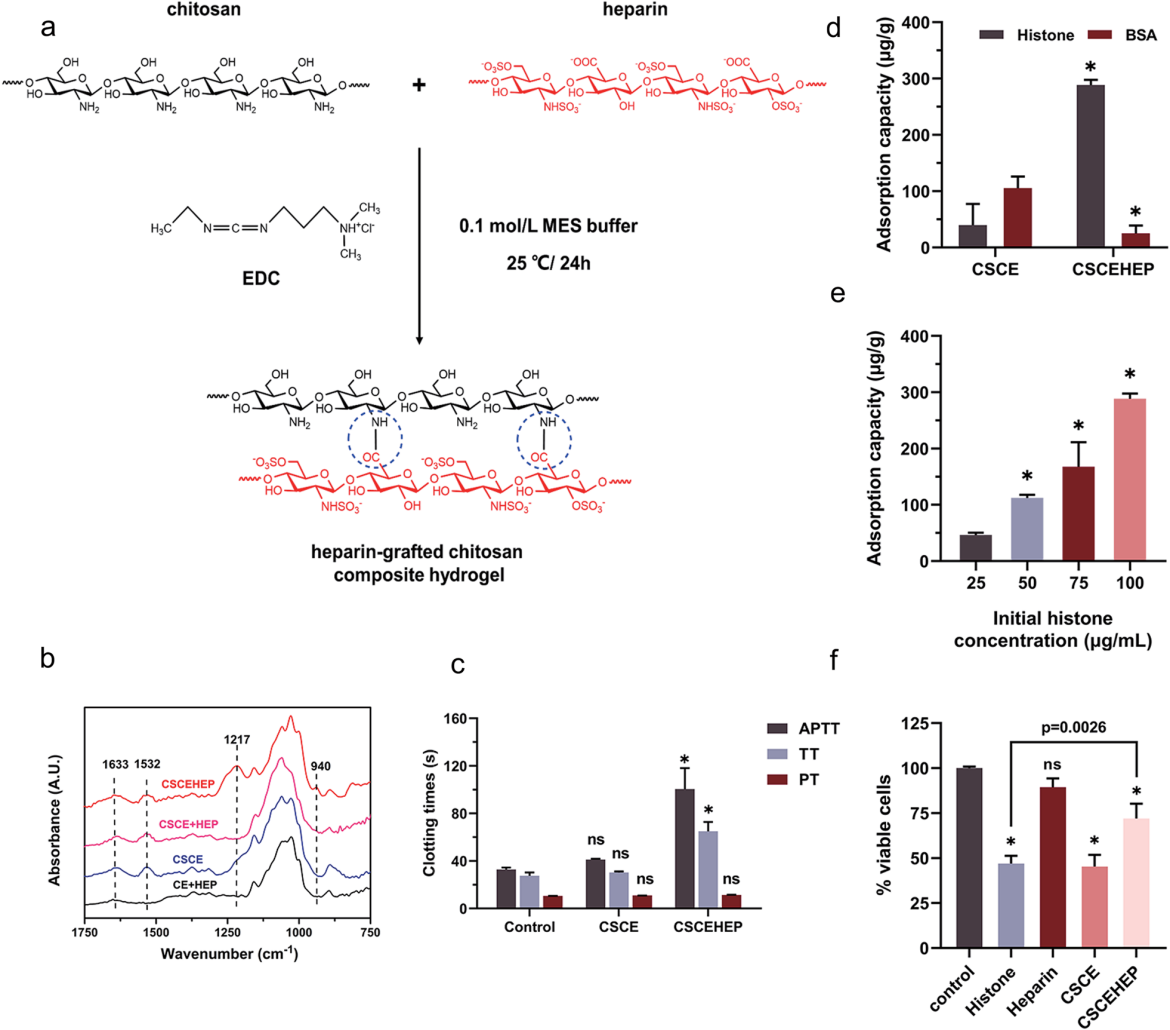
Preparation, characterization and anti-histone properties of heparin-grated chitosan (CSCEHEP) beads.** a** Scheme of the heparin-grafting procedure on chitosan beads; **b** Chemical structure of the CSCEHEP beads determined by Fourier transform infrared spectroscopy; **c** Clotting times of plasma after incubation with the CSCEHEP beads; **d** Histone and BSA adsorption capacities of the CSCEHEP and CSCE beads in batch adsorption experiments; **e** Histone adsorption capacities of the CSCEHEP beads under different histone concentrations; **f** Effect of the CSCEHEP beads on antagonizing histone-induced cytotoxicity ( © 2022 by the authors)

Heparin, a commonly used anticoagulant in clinical practice, has a high negative charge density and a strong affinity for cationic HMGB1 and histones [58, 95]. Using biolayer interferometry, Sharma et al. demonstrated that both unfractionated heparin and low-molecular-weight heparin bind histone subunits with high affinities (Kd < 1 pM-66.7 nM), whereas fondaparinux, a heparinoid, exhibited a low affinity (K_d_ of 3.06 µM-81.1 mM) [96]. Growing evidence has also shown that heparin administration in sepsis models could significantly alleviate the histone-mediated inflammatory response, endothelial dysfunction and lethality [58, 60]. A recent meta-analysis including 2617 participants from 15 RCTs also found that heparin may significantly reduce MODS incidence and 28-d mortality in adult septic patients [97]. However, the increased risk of fatal hemorrhage associated with heparin use in critically ill patients with coagulation disorders remains a major safety concern that limits its wide use in clinical practice. In contrast, extracorporeal hemoperfusion with heparin-functionalized adsorbents may significantly eliminate circulating HMGB1 and histones to exert their immunomodulatory effects. For instance, Marie et al. found that both Seraph-100 and heparin-immobilized Sepharose beads could result in efficient depletion of histone levels in septic plasma samples [35]. Compared to the untreated control, HMGB1 levels were also lowered by both heparin-functionalized adsorbents, resulting in reductions of 58.9 ± 14.7% for Seraph-100 and 63.1 ± 12.2% for heparin Sepharose. More importantly, heparin-functionalized adsorbents could further exert beneficial effects by binding and depleting activated PF4 + platelets and PF4 + platelet extracellular vesicles, thereby contributing to the alleviation of immune thrombosis in sepsis and COVID-19 at multiple levels [35]. Recently, we also developed novel heparin-grated chitosan (CSCEHEP) beads as promising hemoperfusion adsorbents for the selective removal of histones in the blood of septic patients (see Fig. 2) [98]. The results of the batch adsorption experiment showed that the histone adsorption capacity of the CSCEHEP beads increased significantly from 39.9 to 314.3 μg/g, with a reduction rate of 62.8%, compared to that of unmodified chitosan (CSCE) beads. Meanwhile, the CSCEHEP beads significantly antagonized histone-induced cytotoxicity, inflammatory cytokine release and thrombocytopenia. The CSCEHEP beads also prolonged the clotting times of plasma by three times without obvious adverse blood-material reactions, including hemolysis, platelet activation and complement activation, suggesting the satisfactory hemocompatibility of the CSCEHEP beads. Therefore, it is believed that the use of CSCEHEP beads as histone sorbents in EBP sessions may provide novel insight into the modulation of aberrant immune responses in patients with sepsis. Further translational research of these adsorbents in large sepsis animal models is ongoing.

## Conclusions and perspectives

In summary, circulating HMGB1 and histones are the main contributors to inflammation, immune thrombosis, endothelial dysfunction and MODS, which significantly correlate with disease severity and patient-centered outcomes in multiple critical illnesses. Preclinical studies consistently demonstrated that various hemofilters could remarkably eliminate either HMGB1 or histones mainly by the adsorption mechanism, thereby alleviating DAMP-mediated immunopathological effects. However, these encouraging findings should be further confirmed in well-designed clinical trials to determine the optimal initial timing and treatment duration of EBP with such hemofilters, as the precise pharmacokinetics of circulating HMGB1 and histones in critically ill patients remain poorly understood.

Heparin is the most widely used anti-histone agent at present. However, the direct use of heparin in critically ill patients with coagulopathy is contraindicated because the anticoagulant property of heparin will undoubtedly increase the risk of bleeding in these patients. Recently, Sharma et al. found that the ability of heparin to neutralize the cytotoxic and procoagulant effects of histones requires heparin fragments > 1.7 kDa and is independent of the antithrombin-binding pentasaccharide [96], suggesting that non-anticoagulant heparin variants or heparinoids may have therapeutic potential in critical illnesses associated with elevated levels of histones [99–101]. For instance, Karin et al. developed antithrombin affinity-depleted heparin (AADH) by removing the anticoagulant fraction from unfractionated heparin via affinity chromatography. Their results showed that AADH significantly bound histones, prevented histone-mediated cytotoxicity in vitro and reduced mortality from sterile inflammation and sepsis in mouse models [99]. More importantly, AADH treatment of normal mice with a dose five times that of unfractionated heparin caused only moderate anticoagulation of blood plasma and no significant prolongation of tail bleeding time. Similarly, Jine et al. found that a homogeneous chondroitin sulfate E oligosaccharide (CS-E 19-mer) could exert an anti-inflammatory effect by interacting with histones directly and decrease organ damage and mortality in a mouse model of sepsis induced by bacterial lipopolysaccharide without increasing the risk of bleeding [100]. Most recently, the same research group further synthesized a heparan sulfate octadecasaccharide that could not only inhibit the proinflammatory activity of extracellular histone H3 and HMGB1, but also elicit the actions of apolipoprotein A-I to dissociate the complex of HMGB1 and lipopolysaccharide [102]. These encouraging explorations significantly enhance our understanding of the mechanism of action by which non-anticoagulant heparin variants mitigate inflammatory damage and improve survival in sepsis by targeting extracellular histones and HMGB1.

As outlined, hemoperfusion with heparin-functionalized adsorbents also represents an alternative to heparin administration in these critically ill patients to reduce the deleterious effects of HMGB1 and histones. Heparin grafting is a general way to improve the blood compatibility of blood-contacting biomaterials [103, 104], and heparin-grafted membranes are conventionally used in chronic hemodialysis patients at increased risk of hemorrhage [105]. The results of the HepZero study demonstrated that the use of a heparin-grafted membrane is a safe, helpful and easy-to-use method for heparin-free hemodialysis in patients at increased risk of hemorrhage [106]. Similarly, we can speculate that the use of heparin-functionalized adsorbents such as Seraph-100, heparin-immobilized Sepharose beads and our heparin-grated chitosan beads in EBP sessions is theoretically beneficial for the reduction of bleeding risk in critically ill patients.

Beyond heparin-functionalized adsorbents, the direct use of hydrogel-based adsorbents with heparin-mimicking functional groups is also promising for the removal of multiple metabolic wastes by hemoadsorption [104]. For instance, we have successfully developed several self-anticoagulant hydrogel-based adsorbents that share similar structures with heparin in their polymer chains to adsorb endotoxin, bilirubin and low-density lipoprotein from the blood circulation [107–109]. In one of our most recent works, we designed polyethersulfone-based anticoagulant hydrogel microspheres (AHMs) that remarkably bind intrinsic coagulation factors and calcium ions in the extracorporeal circuit to achieve local anticoagulation for heparin-free EBP sessions [110]. These AHMs are composed of a negatively charged copolymer of acrylic acid and 2-acrylamido-2-methyl-1-prroanesulfonic acid. Our preliminary experiment shows that AHMs could significantly adsorb ~ 90% of histones in aqueous solution. Therefore, we speculate that AHMs are promising candidate hemoperfusion sorbents that can modulate dysregulated inflammation, immune thrombosis, endothelial dysfunction and MODS in multiple critical illnesses by adsorbing circulating HMGB1 and histones. Further investigation of the effect of AHMs on eliminating histones and alleviating inflammation in large animal models of sepsis is still ongoing.

It is noteworthy that the removal of circulating HMGB1 and histones might also be associated with potential harm to the human body. For instance, Lange and colleagues demonstrated that HMGB1 significantly contributed to the error-free repair of DNA lesions [111]. Its absence can lead to increased mutagenesis, decreased cell survival and altered chromatin reorganization after DNA damage. More recently, Wu et al. conducted a prospective study enrolling 43 patients with AKI to investigate the association of the removal of DAMPs with mortality in sepsis patients undergoing continuous veno-venous hemofiltration [112]. The results showed that patient survival was significantly worse in groups with higher clearance rates of heat shock protein 70 and HMGB1. Moreover, growing evidence also shows that histones may exert an antimicrobial activity by binding to bacterial nucleic acids and lipopolysaccharides, changing the permeability of the bacterial cell membrane, and inhibiting viral binding and thus playing a vital role in host innate immunity [113]. Therefore, further clinical studies are urgently needed to determine the thresholds of plasma HMGB1 and histone levels for initiating, monitoring or discontinuing EBP with a balanced immunomodulation effect on the innate and adaptive responses. Objective and regular measurements of plasma HMGB1 and histones should also be used to identify homogenous patient populations that benefit most from the elimination of DAMPs. Moreover, the choice of the mortality rate as an endpoint for studies in the ICU setting is potentially fraught with problems. Indeed, a failure to show a survival effect by a certain therapeutic regimen does not simply mean that the regimen is worthless. Future randomized controlled trials should also assess different primary endpoints, such as the number of ventilation-free days, vasopressor therapy-free days, and ICU-free days and the changes of plasma inflammatory mediators, to shed light on other important effects of EBP on critically ill patients [7, 114]. Taken together, the removal of DAMPs during extracorporeal blood purification in critically ill patients with immune dysregulation should be individualized and dynamically changed, and adequate selection of the patient population, treatment dose and type of hemofilters is critically needed.

## Data Availability

Not applicable.
